# Human microRNA expression in sporadic and FAP-associated desmoid tumors and correlation with beta-catenin mutations

**DOI:** 10.18632/oncotarget.16383

**Published:** 2017-03-19

**Authors:** Aldo Cavallini, Maria Teresa Rotelli, Catia Lippolis, Domenico Piscitelli, Rosa Digennaro, Claudia Covelli, Nicola Carella, Matteo Accetturo, Donato Francesco Altomare

**Affiliations:** ^1^ Laboratory of Cellular and Molecular Biology, National Institute for Digestive Diseases, IRCCS “Saverio de Bellis”, Castellana Grotte (BA), Italy; ^2^ General Surgery and Liver Transplantation Unit, Department of Emergency and Organ Transplantation (DETO), University of Bari “Aldo Moro”, Bari, Italy; ^3^ Section of Pathology, Department of Emergency and Organ Transplantation (DETO), University of Bari “Aldo Moro”, Bari, Italy; ^4^ Nephrology Unit, Department of Emergency and Organ Transplantation (DETO), University of Bari “Aldo Moro”, Bari, Italy

**Keywords:** desmoid tumor, microRNA, CTNNB1 mutations, tetraspanin3 (TSPAN3) mRNA, serpin family A member 3 (SERPINA3) mRNA

## Abstract

Desmoid tumors (DT) are rare, benign, fibroblastic neoplasm with challenging histological diagnosis. DTs can occur sporadically or associated with the familial adenomatous polyposis coli (FAP). Most sporadic DTs are associated with β-catenin gene (*CTNNB1*) mutations, while mutated *APC* gene causes FAP disease. microRNAs (miRNAs) are involved in many human carcinogenesis.

The miRNA profile was analyzed by microarray in formalin-fixed, paraffin-embedded (FFPE) specimens of 12 patients (8 sporadic, 4 FAP-associated) and 4 healthy controls. One hundred and one mRNAs resulted dysregulated, of which 98 in sporadic DTs and 8 in FAP-associated DTs, 5 were shared by both tumors. Twenty-six miRNAs were then validated by RT-qPCR in 23 sporadic and 7 FAP-associated DT samples matched with healthy controls. The qPCR method was also used to evaluate the *CTNNB1* mutational status in sporadic DTs. The correlation between sporadic DTs and miRNA expression showed that miR-21-3p increased in mutated versus wild-type DTs, while miR-197-3p was decreased. The mRNA expression of Tetraspanin3 and Serpin family A member 3, as miR-21-3p targets, and L1 Cell Adhesion Molecule, as miR-197-3p target, was also evaluate. *CTNNB1* mutations associated to miRNA dysregulation could affect the genesis and the progression of this disease and help histological diagnosis of sporadic DTs.

## INTRODUCTION

According to WHO (2013), desmoid tumor (DT) belongs to the group of locally aggressive, non-metastasizing mesenchymal tumors and accounts for 0.03% of all neoplasms and 3% of all soft tissue tumors [1].

Although DTs do not metastasize, they are characterized by a tendency to infiltrate and invade the surrounding organs, thus becoming lethal in some cases depending on their anatomical location [2].

Most of DTs occur sporadically and affect young adults, especially females, but in ~5–10% of the cases it is associated with familial adenomatous polyposis (FAP), an autosomal inherited disease, known as Gardner's syndrome [3].

When feasible, surgery remains the first-line treatment, although a significant risk of local recurrence has been reported [4], even after complete surgical resection. In patients who are not candidates to surgery, different therapeutic options could be considered, including radiation or pharmacological therapy [5, 6]. Anti-inflammatory agents, hormonal blockade, cytotoxic chemotherapy and tyrosine kinase inhibitors have all been used, but with inconsistent and unpredictable results [7, 8].

Heterogeneity in clinical and biological behavior and absence of histological/biological markers represent the two hallmarks of DT, which sometimes can lead to misdiagnosis and consequently to unsuitable therapeutic options [5].

Tumorigenesis of DT is a poorly known multistep process involving progressive cell proliferation with a potential role of APC and *CTNNB1* gene mutations.

*APC* is the only gene in which pathogenic variants cause *APC*-associated polyposis conditions, while *CTNNB1* gene mutations characterize the sporadic DTs in around 85% of the cases [9–12].

Several studies report that abnormal expression of β-catenin due to *CNTTB1* gene mutations (especially S45F mutation), in sporadic DTs, may represent a risk factor for recurrence after surgery procedure [13] and could be associated with a poor response to drug treatment [14]. Furthermore, a potential role of CTNNB mutational status has been also proposed as a diagnostic tool for sporadic DT, emphasizing the importance of the role of β-catenin in this tumor [15].

During the past decade, other indisputable factors involved in the complex mechanisms of human tumorigenesis have been identified, including the microRNAs (miRNAs), short non-coding RNA that play a prominent role in a variety of physiologic and pathologic biologic processes, including cancer [16]. However, full analysis of the miRNA profile in DT has not yet been performed.

In this study we analyzed for the first time the mature miRNA profile in formalin-fixed, paraffin-embedded (FFEP) specimens of 30 patients, 23 with sporadic and 7 with FAP-associated DTs, without radiotherapy or drugs pre-treatment and in normal tissue samples of 10 non-DT patients as control.

After evaluation of the CTNNB1 mutational status in sporadic DTs, we correlated the altered miRNA expression between mutated (M) and wild-type (Wt) sporadic DTs.

## RESULTS

### miRNA profile by microarray

One hundred and one of 2080 miRNAs identified by the microarray analysis resulted differently expressed in desmoids tumors compared to normal controls. Five out the 101 miRNAs (miR-409-3p, miR-487b, miR-601, miR-542-5p and miR-4707-5p) were shared by both tumor types, 3 miRNAs (miR-320e, miR-497-5p and miR-2276) were specific to FAP-associated DTs and 93 were specific for sporadic DTs.

Microarray data have been deposited in NCBI's Gene Expression Omnibus and are accessible through GEO Series accession number GSE89687. Supplementary Table 1 summarizes these data.

The IPA algorithm analysis showed that the miRNA targets of the 101 dysregulated miRNAs were part of the bio-functional network group relating to inflammatory diseases, organ injury and connective tissue disorders in sporadic and FAP-associated tumors (Supplementary Figure 1A and 1B, respectively).

### miRNA validation

Comparative qRT-PCR analysis was used to further validate the results obtained from our microarray data.

Subsets of 26 miRNAs were selected for validation, in particular let-7b-3p, let-7f-3p, miR-21-3p, -34a-5p, -133a, -197-3p, -324-5p, -331-3p, -409-3p, -483-3p, -486-5p, -487b, -497-5p, -542-5p, -601, -760, -766-3p, -1281, -3162-3p, -3195, -3651, -4508, -4649-3p, -4707-5p, -4769-3p, and miR-6126.

**Figure 1:**
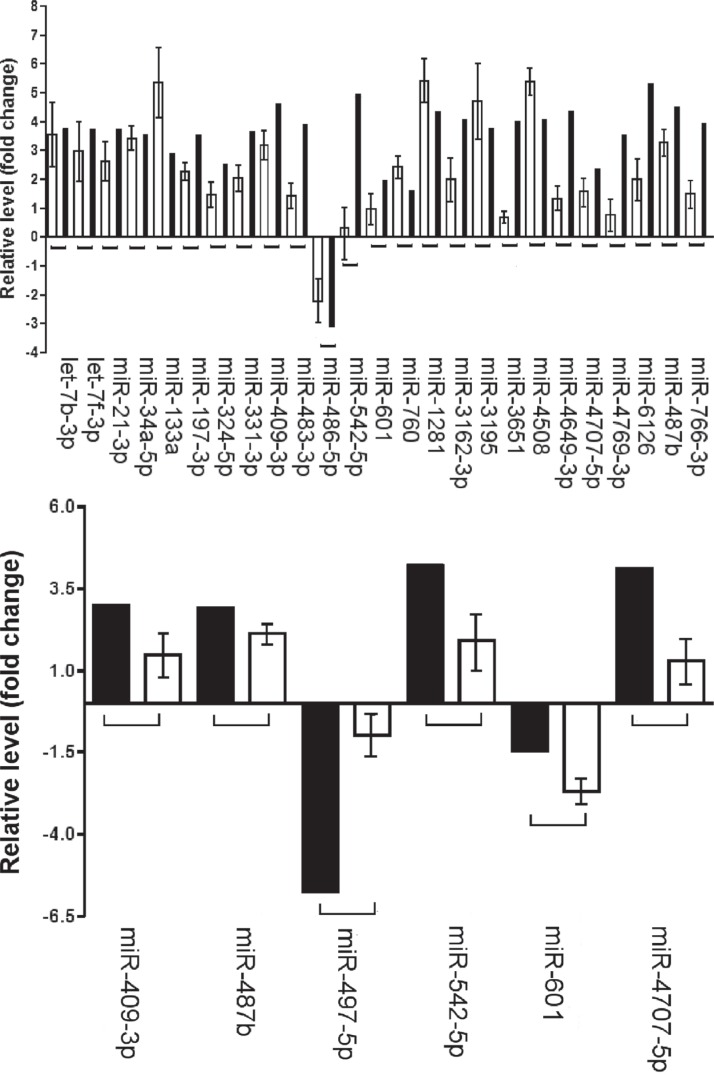
Validation of microarray analysis Comparison between microarray (mean) and RT-qPCR (mean ± SD) data (**A**) Comparison of the miRNA profiling in sporadic desmoid tumors. (**B**) Comparison of the miRNA profiling in FAP-associated desmoid tumors. The Y axis shows the fold change values between tumor samples and controls obtained by microarray and RT-qPCR experiments, respectively.

Twenty of the 26 miRNAs belonged exclusively to the sporadic DT group, 5 miRNAs (miR-409-3p, miR-601, miR-542-5p, miR-487b and miR-4707-5p) were present in both tumor types, while miR-497-5p was detected only in Gardner's syndrome samples (Figure 1A and 1B).

Of these, 23 miRNAs were up-regulated, 2 down-regulated and one, miR-601, showed an opposite trend. It was up-regulated in sporadic tumors and down-regulated in FAP-associated tumors.

Despite consistent mild lower fold change values in few assessed miRNAs by qRT-PCR, a direct correlation was evident between the results obtained in both techniques by Pearson's test (*r* = 0.37; *p* = 0.0003).

### CNTTB1 mutational profile

The exon 3 mutations of the CNTTB1 gene were detected in 14 of 23 sporadic DTs (60.87%).

The type of mutations identified were c.121A > G (p.T41A) in 13 cases and c.134C > T (p.S45F) in only one case.

Expression levels of 25 miRNAs found in sporadic tumors were then compared with the mutational (M) or wild type (Wt) status observed by qPCR analysis.

**Figure 2:**
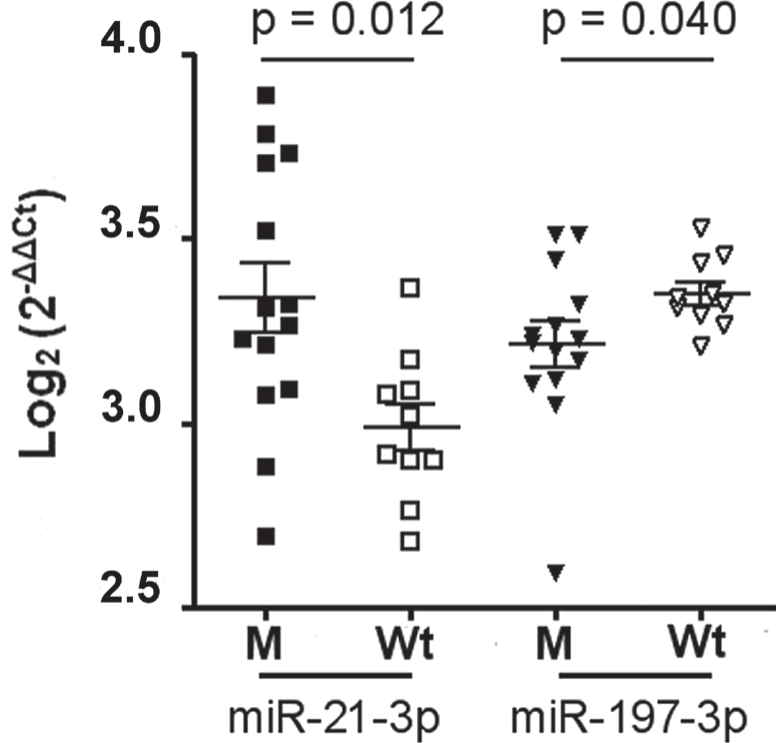
miR-21-3p and mR-197-3p expression in sporadic desmoids with and without *CNTTB1* gene mutations Of 26 patients with sporadic desmoids, 14 showed the gene mutated (M), while the remaining 12 had the wild type gene (Wt). The scatter dot blot graph shows the distribution of each miRNA relative to *CNTTB1* mutational status. The miRNA levels, generated by qRT-PCR, are expressed as log2 (2^−ΔΔCt^) that represents the fold change values of tumor vs. control samples. Differences between two M and Wt groups were evaluated by nonparametric Mann-Whitney test with significant value of *p* < 0.05.

Among them, miR-21-3p and miR-197-3p showed a different distribution between two subgroups (Figure 2). miR-21-3p was more present in M than in Wt group, whereas miR-197-3p was down-regulated in M group.

### Expression of mRNA targets

We selected three mRNAs, Collagen Type I, Alpha 1 (COL1A1), SAM synthetase2 (MAT2A) and L1 cell adhesion molecule (L1CAM), regulated by miR-21-3p, and two miRNAs, Serpin Family member 3 (SERPINA3) and Tetraspanin 3 (TSPAN3), regulated by miR-197-3p by DIANA-TarBase v7.0 database and literature data. miR-21-3p and miR-197-3p are involved in the inflammatory process and pro-fibrotic effect via their mRNA targets [21–24].

The miR-21-3p is a positive regulator for L1CAM and COL1A1 [21, 22] and a negative regulator for MAT2A [23], while SERPINA3 and TSPAN3 are inversely correlated to miR-197-3p expression [24].

The mRNA expression was evaluated by RT-qPCR described above and the list of the primers used are shown in Table 2.

**Table 1:** Clinicopathologic characteristics of desmoid-type fibromatosis with or without associated Gardner fibroma

	Patients (*n* = 30)
**Age (years)**	
Median	39
Range	16–65
**Gender**	
Male	10
Female	20
**Primary surgery**	
Yes	30
No	0
**Pre-operativ therapy**	
Yes	0
No	30
**Tumor size (cm)**	
Median	4.8
Range	2.0–18.0
**Tumor site**	
Intra-abdominal	12
Chest/Abdominal wall	11
Extra-Abdominal	7
**FAP**	
Yes	7
No	23

**Table 2:** List of primers used in RT-qPCR assay for mRNA targets in mutated vs wild type sporadic desmoids

Gene	Gene ID	Sequence 5′ > 3′ F = forward; R = reverse	PCR product
COL1A1	NM_000088	(F) GTGGCCTGCCTGGTGAG(R) GCACCATCATTTCCACGAGC	70 bp
MAT2A	NM_005911	(F) GACAGCTCAACGGCTTCCAC(R) ACAAATCTTATCTGGGTGGCCTT	95 bp
L1CAM	NM_000425	(F) ACACCATGTGATGGAGCCAC(R) GGCTGATGTCATCTGTGGGG	80 bp
SERPINA3	NM_001085	(F) CTCCCTGAGGCAGAGTTGAG(R) TGTTAGGGTGGCAGAGGACA	100 bp
TSPAN3	NM_198902	(F) GGCATCACCTCCTCCAAGAC(R) ATAAAATGCCAGCTGCCCCC	70 bp
β-actin	NM_001101	(F) AAAGACCTGTACGCCAACACAGTGCTGTCTGG(R) CGTCATACTCCTGCTTGC TGATCCACATCTGC	220 bp

**Figure 3:**
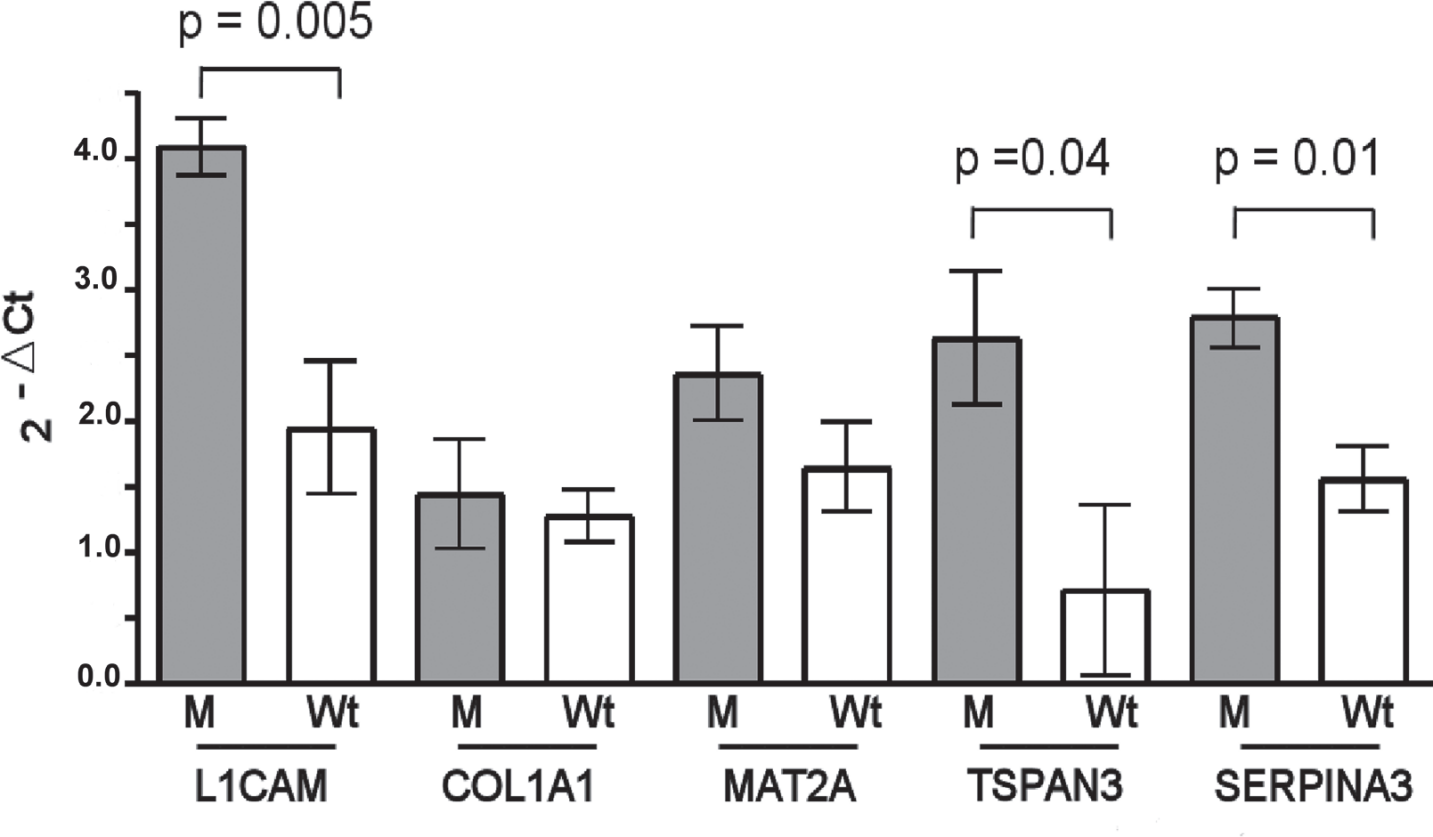
Expression of the miR-21-3p and miR-197-3p mRNA targets miR-21-3p promotes the L1CAM expression, while the negative regulation for COL1A1 and MAT2A is not observed in mutated (M) vs. wild type (Wt) sporadic desmoid tumors. Decreased levels of miR-197-3p induce a TSPAN3 and SEPINA3 mRNA over-expression in M vs. Wt sporadic tumors. The gene expression was quantified using RT-qPCR. Bars represent the mean ± SD and the values are significant for *p* < 0.05.

We found that the *L1CAM, TSPAN3* and *SERPINA3* gene expression were significantly up-regulated in M than Wt sporadic tumor samples, whereas COL1A1 and MAT2A mRNA showed similar levels in both groups (Figure 3).

### Immunohistochemical analysis for β-catenin

As reported in Table 3, β-catenin protein was expressed at the nuclear level in patients with sporadic tumor carrying the T41A mutation and in only a Wt tumor case.

**Table 3:** Nuclear expression of the β-catenin proteins in 23 sporadic DTs with and without mutations in *CTNNB1* gene

	β-catenin protein expression				
Mutation type	N°	−	+	++	++++
T41A	13	1	5	7	0
S45F	1	1			
Wild-type	9	8	1	0	0

Score system: 0–5% of cells (negative, negative symbol); 6–25% (weakly positive, positive symbol); 26–50% (moderately positive, double positive symbol); > 50% (strongly positive, triple positive symbol).

Five and seven tumor samples with T41A mutation showed a weak and moderate positivity, respectively, for nuclear β-catenin proteins, whereas the one tumor sample with S45F mutation does not show the β-catenin proteins in the nucleus.

## DISCUSSION

Recent studies have clearly demonstrated the relevance of miRNAs in regulating the cellular metabolism in several human diseases, but their role in the desmoid tumor remains substantially unexplored.

To our knowledge, this is the first study concerning the miRNA profile in both sporadic and FAP-associated desmoid tumors of patients before any medical therapy.

The microarray analysis revealed a great number of miRNAs differently expressed in these two tumor types compared to controls, suggesting a deep molecular heterogeneity among them, despite their histological similarity.

About 90% of dysregulated miRNAs were present in sporadic DTs and only 10% were associated to the Gardner's syndrome. Furthermore, the bioinformatic analysis showed several biological functions linked to these miRNAs, as cell proliferation and differentiation, response to the inflammatory process and tumor growth.

A good correlation was observed between RT-qPCR and microarray analysis. All selected miRNAs showed similar trends.

The analysis of the *CTNNB1* mutational status, gene codifying β-catenin protein, shows the presence of mutations in about 60% of the sporadic DTs (14 out of 23 patients). Furthermore, the immunohistochemical analysis confirmed the nuclear location and accumulation of the β-catenin proteins in mutated sporadic DTs.

These data are in agreement with the literature. In fact, *CTNNB1* gene mutations in the sporadic DTs is reported to occur in 60–85% of the cases [9–13].

The occurrence of these mutations seems to have clinical relevance, the DT recurrence after surgery seems to be strongly associated with p.S45F mutation [13], while patients with wild-type tumors seem to have better prognosis [9, 10, 12, 13].

A limitation of our study is the inability to correlate the different mutations with clinical features due to the limited number of patients considered.

However, it is worth to note the high prevalence of p.T41A mutation in our cohort of patients with sporadic DTs is in agreement with other authors [9–12].

Since the high frequency of mutations in the *CTNBB1* gene characterizes sporadic DTs, the analysis of the mutational status of this gene has been suggested as a useful tool in the differential diagnosis with other soft tumors [15, 16].

Our RT-qPCR data showed that miR-21-3p and miR-197-3p were significantly altered in sporadic mutated as compared to Wt DTs. The over-expression of the miR-21-3p was associated to high levels of nuclear β-catenin protein in mutated DTs.

These results are consistent with the *in vitro* experiments reported by Lin et al. [25] who demonstrated that over-expressed miR-21-5p (opposite arm of the miR-21-3p) in colorectal cancer cell lines promoted β-catenin nuclear translocation via increased phosphorylation of this protein at Serine552 and this phenomenon was associated with mutated *CTNNB1* gene.

Moreover, Veronese et al. [26] observed the same cellular mechanism described by Lin et al. for β-catenin negative regulator miR-483-3p. All these data strengthen the concept that mutated *CTNNB1* gene produces a phosphorylated form of the β-catenin that evades the miRNA regulatory effect. The same cell-cycle dysregulation may be conceivable in sporadic DTs that show mutated *CTNNB1* gene. The finding of dysregulated miR-197-3p and miR-21-3p expression, in combination with the mutational analysis of the CTNNB1 gene, could also be used by pathologists as an additional tool for a correct diagnosis in case of unclear pathological assignment.

In order to generate miRNA-mRNA interactions, we have examined some mRNA targets of these two miRNAs.

However, it must emphasize that our study was an exploratory study and a limited number of target genes was considered although there are many additional target genes that warrant further investigation.

We focused on three mRNA targets (COL1A1, MAT2A and L1CAM ) of the miR-21-3p, and, among them, only L1CAM showed an aberrant expression in the mutated sporadic DTs in association to the increased levels of miR-21-3p.

In other human tissues, L1CAM promoter is activated by Wnt/β-catenin signalling pathway and nuclear β-catenin proteins [27]. Furthermore, L1CAM expression, positively regulated by miR-21-3p, regulates IL-1β over-expression that in turn is also a classical NFkB inducer [28].

Taken together these findings suggest that increased miR-21-3p levels, with consequent increase of L1CAM proteins, associated to a nuclear location of the β-catenin proteins could contribute to a pro-fibrotic alteration, as well as a pro-inflammatory regulation, in a subset of sporadic DTs showing *CTNNB1* gene mutations.

The miR-197-3p role in the tumors is not yet fully understood. However, in pancreatic cancer cells miR-197 act as a negative regulator of the delta-catenin, an adhesive junction protein of the armadillo/beta-catenin superfamily [29].

To further explore the role of the reduced expression of miR-197-3p in mutated sporadic DT, we investigated the expression of two target genes, SERPINA3 and TSPAN3, that are negatively regulated by miR-197-3p [24].

We found that gene expression of both SERPINA3 and TSPAN3 was increased in mutated tumors.

Although the physiological function of the SERPINA3 and TSPAN3 proteins in sporadic DTs is unknown, in HCC cell model the inflammatory pathway interferes with mature miR-197-3p biosynthesis [30] and, in particular, increased SERPINA3 levels were associated to the acute and chronic inflammation [31].

Therefore, over-expression of miR-21-3p and down-expression of miR-197-3p targeting L1CAM, SERPINA3 and TSPAN3, respectively, could induce or maintain the inflammatory process in mutated desmoid tumor.

In conclusion, we describe, for the first time, the miRNA profile in desmoid tumors supporting the concept that their dysregulation might be involved in the oncogenesis and progression of this disease, and, despite the consideration that further confirms are needed before to consider a single miRNA or a miRNA family as possible targets for future therapeutic strategies in patients with sporadic DTs. These findings may support the role of mRNA as future biomarker [32] and onco-target therapies against this insidious disease [33].

Furthermore, our data suggest that the analysis of the miR-197-3p and miR-21-3p expression, in combination with the mutational analysis of the CTNNB1 gene, could be today a useful tool for a correct diagnosis of sporadic DTs.

## MATERIALS AND METHODS

### Patients and controls

Thirty consecutive patients with desmoids, submitted to surgery in General Surgery and Liver Transplantation Unit between 1999 and 2015, entered the study. Patients with previous history of cancer, recurrent DT, pretreatment with non-steroidal anti-inflammatory drugs, chemotherapy, hormonotherapy or radiotherapy were excluded.

There were 23 patients with sporadic DTs, while 7 had a FAP-associated DTs.

Clinical and demographic data of the patients are shown in Table 1.

As control, normal connective tissue specimens were obtained from the fascia of 10 patients (8 males and 2 females, median age 45 years, range 16–65 years) who underwent surgery for inguinal hernia in General Surgery and Liver Transplantation Unit.

These patients were chosen because affected by non-inflammatory and non-tumoral diseases.

Informed consent was obtained from all patients at the time of surgery and the study was approved by Institutional Ethics Committee of University of Bari, Italy (n.5038/16).

### Sample collection

The study was carried out on formalin-fixed paraffin-embedded (FFPE) tumor specimens of the DTs obtained by the Pathology Department.

Consecutive FFPE tissue sections (4-μm thick) from the same block of each patient were cut for miRNAs, mRNAs, DNA mutations and immunohistochemical analysis.

The control samples were obtained during the hernioplasty and a sample of fibrous tissue measuring 2.0 × 0.5 cm was taken from the fascia of the conjoint tendon of the inguinal canal. After fixation and paraffin method, the samples were analyzed by pathologist. These tissues showed a connective structure highly differentiated and poorly cellular, formed from mature fibroblasts intermingled and surrounded by tightly packed collagen fibers. Such histological features are similar to those seen in DTs.

### Nucleic acid isolation

Total RNA and DNA were isolated from FFPE tissue samples by miRNeasy FFPE Kit and miRNeasy mini Kit (Qiagen, Milan, Italy). All procedures were performed according to manufacturer's protocols.

The yield and quality of RNA was determined by Agilent 2100 Bioanalyzer instrument (Agilent Technologies, Santa Clara, CA, USA). All RNA and DNA samples were stored at −80°C until analysis.

### Mature miRNA profiling by microarray

To define a specific miRNA profile, we performed miRNA microarray analysis (2080 mature miRNAs) on FFPE tissue samples of 8 sporadic DTs, 4 FAP-associated DTs, regardless to tumor site, and 4 control samples. Microarray steps were carried out according to the manufacturer's protocol.

Normalization was performed according to the Quantile method. The selection of the differentially expressed probes between tumor and control samples was performed applying an unpaired t-test, with a p-value cut-off of 0.05 and a fold change (FC) cut-off of 2.

Ingenuity Pathways Analysis software (IPA, Qiagen, Redwood City, CA, USA), that embraces a large database of biological and functional relationships was used to interpret the microarray data [17].

### Microarray validation

To validate the microarray data by RT-qPCR method previously described [18], we have chosen 26 miRNAs of both FAP-associated and sporadic DTs that were mainly involved in Wnt/β-catenin signalling pathway or proliferation process. Twenty five miRNAs had levels of log_2_ fold-change lower or higher as compared to those of control group (for details, see Supplementary Table 1), whereas miR-601 was chosen because this miRNA showed a different trend in two DT types. It was down-expressed in FAP-associated DTs and over-expressed in sporadic DTs.

The miRNA expression was calculated by 2^−ΔΔCt^ method, where ΔΔCt = ΔCt_(miRNA target)_ - ΔCt_(mean value of the controls)_, corresponding to FC value of each miRNA in DT vs control samples.

The RT-qPCR and microarray FC values were transformed to log2 values and compared with each other by Pearson's correlation to determine at what frequencies the general trends of up- and down-regulation were conserved in both methods used.

### CNTTB1 gene mutations in sporadic tumors

The DNA of the sporadic DTs was screened to evaluate the presence of the three most frequent mutations of the *CNTTB1* gene (*c.121A > G* (p.T41A), *c.134C > T* (p.S45F) and *c.133T > C* (p.S45P)).

The *CNTTB1* gene mutations were detected by three qBiomarker Somatic Mutation PCR Assay kits (catalog # SMPH003987A, # SMPH003953A and # SMPH003970A for 121A > G, 134C > T and 133T > C mutation, respectively) (Qiagen) according to manufacturer's protocol.

The PCR assay, based on ARMS technology, was run in triplicate for each sample and appropriate positive (sporadic colorectal carcinomas with microsatellite instability) and negative (surrounding “normal” tissue of the colorectal carcinomas) control samples were used in each batch.

Different expression of the validated miRNAs between mutated (M) and wild type (Wt) sporadic DTs was also evaluated.

### RT-qPCR for mRNA expression

The mRNA targets of the miRNAs differentially expressed in M and Wt sporadic DTs were also analyzed.

These mRNAs were selected by Diana-TarBase v7.0, a public database showing miRNA:mRNA interactions experimentally validated [19].

The RT-qPCR procedures were previously described [20]. All PCR were carried out in triplicate using cDNA synthesized from the same batch and starting from same amount of total RNA. Negative controls containing no cDNA template were included for single gene within each PCR run. The relative mRNA levels were determined by 2^−ΔCt^ method, where ΔCt = Ct_(mRNA target)_ - Ct_(β-actin)_.

### Immunohistochemical analysis

After collection, desmoids tumor samples were fixed in formalin, embedded in paraffin and consecutively sectioned at 4 um thickness.

Hematoxylin and eosin staining has been performed for each sample and reviewed by the pathologist.

Following deparaffinization, antigen retrieval and endogenous peroxidase blocking, sections were incubated with rabbit monoclonal beta-catenin antibody for 20 min at RT ( 1:100 dilution, Thermo Fisher Scientific, Milan, Italy) in order to perform the immunoistochemical analysis for localization (nuclear or cytoplasmatic) and quantization of the β-catenin proteins in M and Wt sporadic tumors. Bound antibody was detected by Envision Flex Mini kit and DAKO autostainer instrument (Dako Italia, Cernusco sul Naviglio (MI), Italy).

The 4-tiered scoring system, based on the positive percentage of counted cells (0–5% negative or “-“ ; 6–25% weakly positive or “+” ; 26–50% moderately positive or “++”; > 50% strongly positive or “+++”) was used for quantitative evaluation [26]. One hundred cells from 5 different fields for each sample, were randomly selected and counted by two independent observers. A third independent pathologist served to reach a final consensus in contrasting evaluations.

### Statistical analysis

Data obtained by both 2^−ΔCT^ and 2^−ΔΔCT^ methods were expressed as mean ± SD.

Pearson's correlation was used for the comparison of RT-qPCR and microarray data expressed as log_2_ FC.

The relationship of the miRNAs between M and Wt sporadic DTs were analyzed using Mann-Whitney test and *p*-value < 0.05 was considered significant.

For statistical test, GraphPad Prism 5 (San Diego, CA, USA) was used.

GeneSpringGx12.5 was used for microarray analysis

## SUPPLEMENTARY FIGURE AND TABLE




