# Supplementary material for: Human microRNA expression in sporadic and FAP-associated desmoid tumors and correlation with beta-catenin mutations
Source: Oncotarget. 2017 Mar 19;8(26):41866–75. doi: 10.18632/oncotarget.16383 (PMC5522034; doi:10.18632/oncotarget.16383)
Supplement: Supplementary file 1 [file oncotarget-08-41866-s001.pdf]

Human microRNA expression in sporadic and FAP-associated desmoid tumors and correlation with beta-catenin mutations

Supplementary Materials

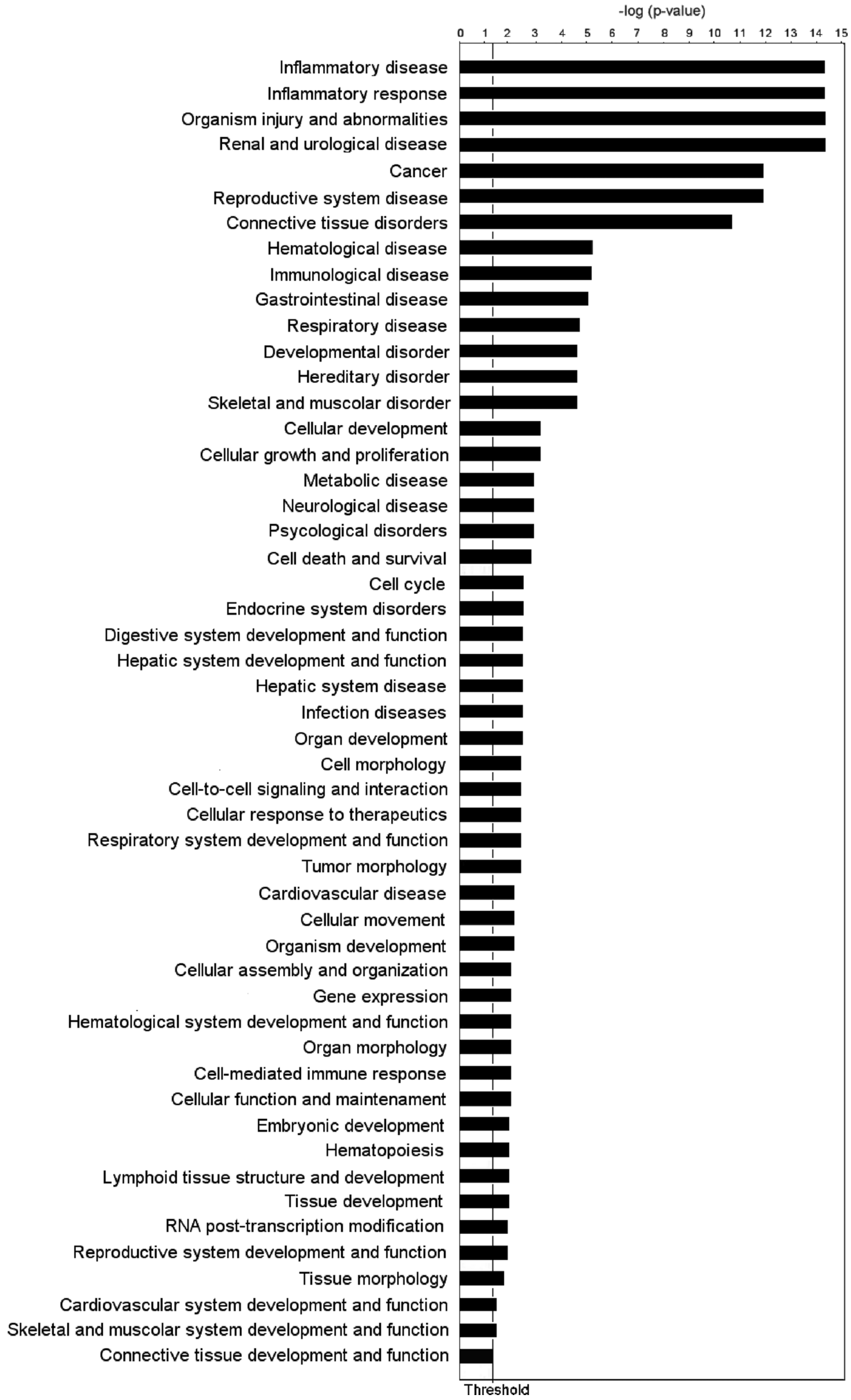

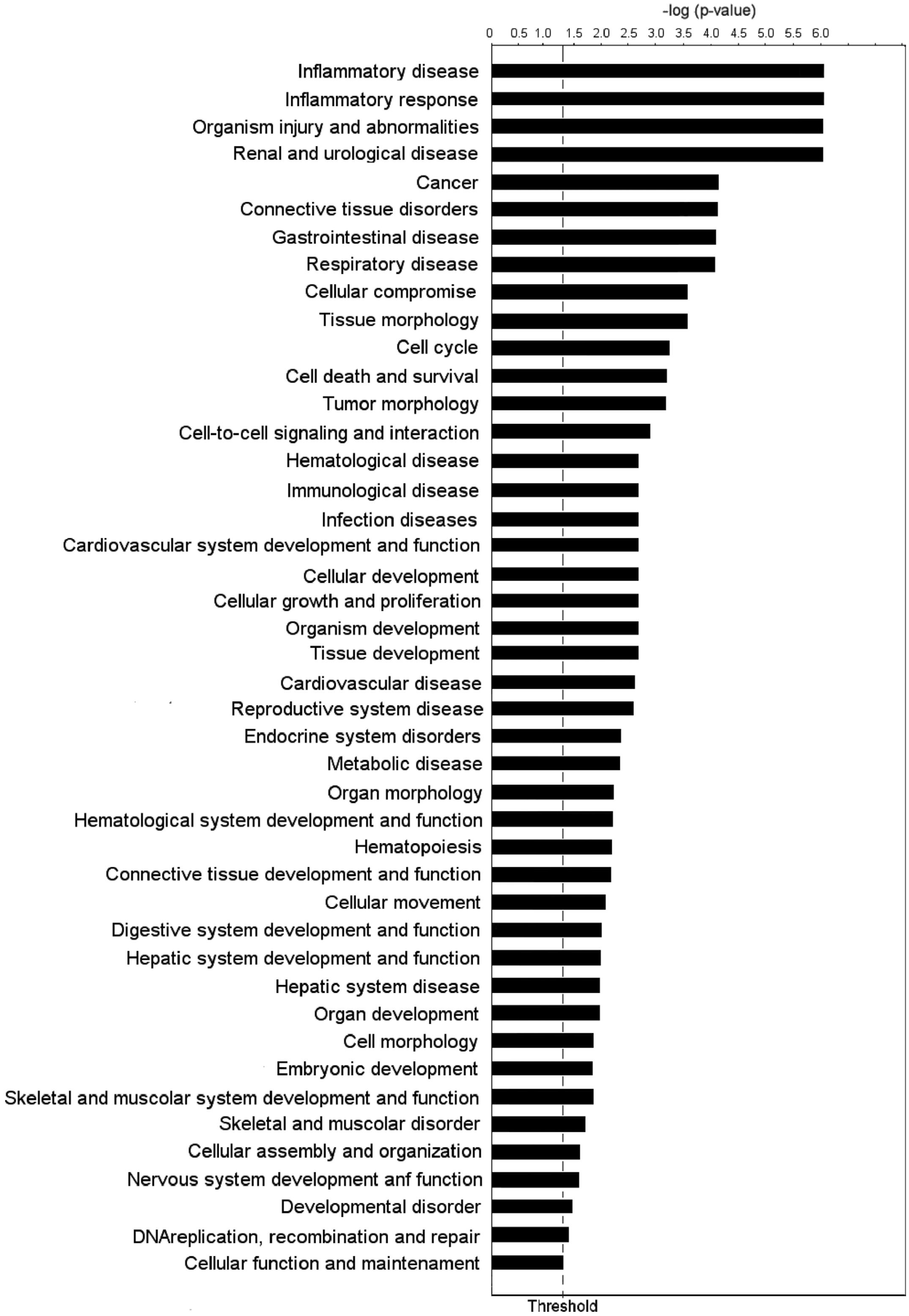

Supplementary Figure 1: Ingenuity pathways (IPA) analysis. Functional classification of the dysregulated miRNAs found by microarray analysis was identified by Ingenuity Pathway Analysis software in sporadic (**A**) and FAP-associated (**B**) desmoid tumors. The vertical axis represents biological functions and diseases, while the horizontal axis represents the log(p) value for each function/disease. The threshold line corresponds to $p < 0.05$. The figure has been changed in a gray scale than the original for better reading.

Supplementary Table 1: miRNA profile by microarray analisis (desmoids tumors vs controls). See Supplementary_Table_1
